# Evaluating implementation outcomes (acceptability, adoption, and feasibility) of two initiatives to improve the medication prior authorization process

**DOI:** 10.1186/s12913-021-07287-2

**Published:** 2021-11-20

**Authors:** Laney K. Jones, Ilene G. Ladd, Christina Gregor, Michael A. Evans, Jove Graham, Michael R. Gionfriddo

**Affiliations:** Geisinger Health System, 100 N Academy Avenue, Danville, PA 17822 USA

**Keywords:** Implementation science, Prior authorization, Qualitative

## Abstract

**Background:**

Processes such as prior authorization (PA) for medications, implemented by health insurance companies to ensure that safe, appropriate, cost-effective, and evidence-based care is provided to all members, have created inefficiencies within healthcare systems. Thus, healthcare systems have implemented supplemental processes to reduce burden and ensure efficiency, timeliness, and appropriate care.

**Objective:**

Evaluate implementation outcomes of two initiatives related to PA for medications: a common record that records all PA-related information that was integrated into the health record and an auto-routing of specialty prescriptions to a hospital-owned specialty pharmacy.

**Methods:**

We conducted semi-structured interviews with medical staff to understand their experience, acceptability, adoption, and feasibility of these initiatives guided by Proctor’s Framework for Implementation Outcomes. Transcripts were analyzed using consensus coding.

**Results:**

Eleven medical staff participated in semi-structured interviews. The two initiatives were analyzed together because the findings were similar across both for our outcomes of acceptability, adoption, and feasibility. Participants found the implemented initiatives to be acceptable and beneficial but felt there were still challenges with the new workflow. The initiatives were fully adopted by only one clinic site within the healthcare system, but limitations arose when adopting to another site. Individuals felt the initiatives were feasible and improved workflow, communication, and transparency. However, participants described future adaptations that would help improve this process including improved standardization, automation, and transparency.

**Conclusion:**

The acceptability, adoption, and feasibility of two initiatives to improve the PA process within the one clinical site were well received but issues of generalizability limited the initiatives adoption system wide.

**Supplementary Information:**

The online version contains supplementary material available at 10.1186/s12913-021-07287-2.

## Introduction

Insurance companies have implemented processes to ensure safe, appropriate, cost-effective, and evidence-based care is provided to their members. One of these processes is prior authorization (PA) which stipulates a unique set of requirements to be completed before a medication is dispensed or a procedure occurs. The decision-making process and evidence used to assess the appropriateness of therapy as part of PA differs by insurance company [1]. The exact process and reasons for decisions are often not transparent for patients or their clinicians. This process is often seen as burdensome by patients and clinicians and may lead to treatment delays, administrative costs, and workflow burden. However, the literature on this topic is mixed with some studies showing a reduction in cost for patients [2] while others have shown high costs for patients and providers [3, 4]. Additionally, there has also been mixed reports of impact on patient outcomes [2, 5].

Members of the healthcare team must complete these requirements, submit documentation to the insurance company, and receive an approval or denial letter. Every healthcare entity has operationalized workflows for how to handle PAs within their facility, and these workflows can potentially be time consuming and burdensome in many healthcare systems [6]. For example, some systems have dedicated front office staff that solely work on PA for that particular clinic, while others have implemented teams of individuals whose only responsibility is to complete and submit PA requests for any patient within the healthcare system. One reason for the time and burden associated with PA is that requirements can differ within insurance products from one company and between insurance companies [1]. Thus, healthcare systems need more efficient systems to handle workflow and communication about PAs and their status to any potential healthcare team member so that patients can receive safe, appropriate, cost-effective, and evidence-based care in a timely manner.

Previous work in our healthcare system found that major barriers in the PA process included the lack of standardized process including multiple documentation forms and workflows [1, 7]. To address these limitations, we embarked on two initiatives to streamline PA in our system: developing a common record for PA within the electronic health record (EHR) and introducing a standardized routing process for specialty medications. This paper describes medical staff’s experience implementing these initiatives, focusing on the implementation outcomes of acceptability, adoption, and feasibility.

## Methods

### Setting

Geisinger is an integrated delivery system that serves 45 counties in Pennsylvania, with 6 hospital campuses and a network of 138 primary and specialty clinic sites including 46 primary care sites, 20 convenient care sites (urgent care), two “65 forward” sites (primary care for seniors), 2 community care sites, and 4 LIFE Geisinger sites (program of all-inclusive care for the elderly). Geisinger utilizes a single EHR in all hospitals and clinics throughout the entire system, and it owns and operates multiple retail pharmacies as well as a specialty pharmacy. In addition, Geisinger has a department of individuals that specialize in submitting PAs.

### Initiatives

A system-wide initiative to improve the transparency, documentation, and care for patients prescribed medications that required a PA from their insurance company was undertaken. A clinical team of high utilizers of PAs requests for their patients worked with the quality improvement team to understand the current workflow and to develop and test initiatives to improve current workflow inefficiencies from the healthcare system. This team developed two initiatives: 1) a common record and 2) an auto-referral of specialty medications to a specialty pharmacy.

The first initiative was the development of the common record. The quality improvement team identified three major workflow inefficiencies related to the healthcare systems’ PA workflow: 1) there was not a spot within the EHR to document information related to the status of a PA for a specific medication, 2) the workflow for documentation of the PA information differed between hospital/clinic sites and within departments of the healthcare system, and 3) information required for the PA was not accessible to all team members. The purpose of the common record was to allow for a single source of documentation in the EHR for all information related to PA that would be accessible to all members of the healthcare team. This information includes medication insurance type, medication, approval status, out of pocket costs, availability of financial assistance. The structure of the common record was a single document within the EHR that was editable for all team members that worked on the PA. Previously, individuals that worked on the PA for a patient would communicate via telephone encounters which are only available to the individuals attached to the thread and do not become a part of the EHR of the patient. A more detailed description of the common record workflow is described in Table 1. Departments that had a stake in the PA workflow had at least one representative on the internal team tasked to develop the common record. The common record was piloted in one specialty clinic location in the healthcare system. Due to the complexity of the PA workflow and problems that arose with this pilot, the common record was not initiated system-wide; thus, different workflow existed for processing PA at the time of analysis.

**Table 1:** Description of components of the initiatives to improve the process for completion of medication PA at the healthcare system

Domain	Initiative: Common record	Initiative: Auto-referral
Actor(s)	*- Clinician (*e.g. *physician, advanced practitioner)* *- Front office staff* *- Department that specializes in completing PA requests* *- Pharmacists at the hospital-owned specialty pharmacy*	*- Clinician (*e.g. *physician, advanced practitioner)* *- Pharmacists at the hospital-owned specialty pharmacy* *- EHR*
Action(s)	*- Clinician (*e.g. *physician, advanced practitioner)* – orders a medication that requires PA and sends it to a pharmacy *- Front office staff* – Receives request from clinician regarding PA request for a medication and initiates the common record *- Department that specializes in completing PA requests* – Gathers necessary clinical and insurance information to complete the PA request, submits the PA request, and documents within the EHR *- Pharmacists at the hospital-owned specialty pharmacy* – Pharmacy receives the medication request and fills and dispenses the medication to the patients after the completion of the PA	*- Clinician (*e.g. *physician, advanced practitioner)* – orders a medication that requires PA and sends it to a pharmacy *- Pharmacists at the hospital-owned specialty pharmacy* – Pharmacy receives an auto-referral for the prescription (if a specialty medication) to the specialty pharmacy when the clinician orders the medication. If the hospital-owned specialty pharmacy can fill the prescribed medication, they will fill it and dispense it to the patient. The ability to fill a medication is dictated by the patient’s insurance company. If they cannot fill the medication, the hospital-owned specialty pharmacy will send the medication to the appropriate pharmacy based on their insurance. *- EHR* – within the medication ordering system, the health record will forward the prescription directly to the hospital-owned specialty pharmacy for a certain list of medications
Target(s) of the action	Patient receives prescribed medication for their condition	Patient receives prescribed medication for their condition
Temporality	Common record is initiated every time the prescribed orders a medication requiring PA	Every medication that is ordered by a clinician that is on the auto-referral list
Dose	All actors should complete their required pieces when available	All clinicians have to use this process
Implementation outcome(s) affected	Uptake of all involved PA staff, adoption of the new documentation process, fidelity to the new process	Uptake of the specialty pharmacy to fill the medication or forward to another specialty pharmacy the medication
Justification	Quality improvement team identified there was no single record of this information within the EHR	Quality improvement team found that we were not filling many of the specialty medications ordered by our clinicians and that we could provide improved care to our patients if we were able to fill these medications

The second initiative aimed to improve the continuity of care for patients seen at Geisinger and involved a predetermined list of medications that would be automatically sent to the Geisinger specialty pharmacy regardless of the pharmacy selected by the patient or provider during the clinic visit. The decision to *auto-route* prescriptions was made because many medications were being filled by non-Geisinger owned pharmacies, when they could be filled at an internal specialty pharmacy. This process allowed for greater continuity of care as the Geisinger specialty pharmacist, due to access to the EHR and relationships with clinics, could monitor the patient for potential adverse reactions and communicate with the healthcare team better than an outside pharmacist. However, the filling of specialty medications is often dedicated by a patients’ insurance coverage and if it was deemed that the prescription could not be filled at the internal pharmacy then it was sent to the appropriate pharmacy. As part of this process, Geisinger’s specialty pharmacy completes PA requests for patients and documents this information within the common record in the EHR. The specialty pharmacy team was notified that this process had been turned on and was trained in the new workflow. A more detailed description of the auto-routing of prescriptions is described in Table 1.

### Study design

This study evaluated the implementation outcomes (acceptability, adoption, and feasibility) of the two initiatives aimed at improving the PA process within the healthcare system. Implementation outcomes were defined using Proctor’s Conceptual Framework for Implementation Outcomes [8]. Implementation outcomes include acceptability, adoption, appropriateness, feasibility, cost, fidelity, penetration, and sustainability which are measured at different time points of the implementation process. We defined acceptability as satisfaction with the initiatives, adoption as the uptake and utilization of the initiatives, and feasibility as the actual fit and practicability of the initiatives. The manuscript reporting was guided by standards for reporting qualitative research [9].

### Procedure

We invited medical staff who work with PA requests and were targeted by at least one of the initiatives to participate in semi-structured interviews. These individuals included providers, front office staff, members of a department that specializes in completing PA requests, and specialty pharmacists; Tables 1 and 2 provide definitions of their roles for medication PAs. The purpose of these interviews was to understand their experience with the implementation of the initiatives to improve the PA process. Interview guides were tailored to the role of the interviewee and included questions related to the implementation outcomes of the initiatives (see Supplemental Material). Two study personnel with qualitative training and no previous interaction with interviewees conducted semi-structured interviews that were audio-recorded and lasted approximately a half an hour. All participants provided verbal informed consented and were assigned a study number for privacy. All methods were carried out in accordance with relevant guidelines and regulations for human subjects.

**Table 2:** Coding tree

Codes	Theme	Description
Perceived benefit	Acceptability	Participants felt both initiatives could be beneficial, but satisfaction was limited due to implementation challenges.
No standardized process Training	Adoption	Adoption was limited due to an ineffective training processes and problems with adaptability of the initiatives.
Workload	Feasibility	Overall, the initiatives were not feasible due to issues related to adaptability resulting in an inefficient process that included duplicative work.
Suggestions	Future adaptations	Participants had several suggestions for improvement including improving: standardization, transparency, and automation.

### Analysis

Interviews were transcribed verbatim, and an initial codebook based on questions from the interview guide was made by two study team members and then mapped to Proctor’s outcomes using inductive analysis [10]. Two independent reviewers with experience in qualitative research and pharmacy coded each transcript and consensus coding was used to resolve any discrepancies. Themes related to acceptability, adoption, and feasibility were then iteratively and collaboratively developed based on the consensus coding. Atlas.ti software was used to facilitate analysis (Version 8.0, Berlin, Germany). This study was approved by the Geisinger’s Institutional Review Board.

## Results

We interviewed 11 participants affected by one or both implemented initiatives: physicians (3), office staff (1), PA experts (3), and pharmacists (4). The majority (8/11) were female and had been in their current roles between 3 months and 32 years. We report acceptability, adoption, and feasibility associated with the implementation framework and future adaptations to the process that were discussed by participants (Table 2). We failed to identify meaning differences between the stakeholder groups responses; thus, there results were analyzed together.

### Acceptability

Participants had an overall positive experience with both implemented PA initiatives. Participants noted that implementation of the common record was a great idea because it created: a standardized workflow, a single location where all PA information could be documented, and a process for pharmacists to access patients’ medication benefit information resulting in less confusion when submitting PA requests. As one participant stated: *“I get it as a single message, and it goes on that. Obviously if everything is kept together, it’s easier to follow”* (Participant #12). Another indicated that: *“I think it [common record] has helped alleviate the back and forth miscommunication”* (Participant #2). This resulted in a situation where ideally, as one participant described: *“[these initiatives] improve communication, it’s better overall in term[s] of efficiency for [our healthcare system] and for the patient”* (Participant #12). However, the complexity of medication PA led to dissatisfaction with the delivery and implementation of the initiatives.

Regarding the auto referral process, providers felt the added value in the service provided by the specialty pharmacy due to the increased touch points with patients and regular follow-up for side effects and adherence to medication. Physicians also mentioned that the specialty pharmacy initiative improved the continuity of care with patients, and others described the financial benefits. For example, a specialty pharmacist stated, *“[W] e increased our business … and the whole continuum of care stays within Geisinger. So, I think that’s a great thing”* (Participant #23).

### Adoption

While the auto-referral process was adopted system-wide, the common record was only adopted at one clinic site.

Adoption was affected by a lack of effective training for both initiatives. Some participants could not recall any or minimal (e.g., an e-mail) training or notification of these initiatives, while others recalled several activities: *“[We told] the staff that this was happening. Fast facts were emailed to all of pharmacy …*. *And a [best practice alert] fired for providers. So, my staff was given all of those tools to learn and understand, so that they could guide providers through the process”* (Participant #26). This lack of training and support may have led to the situation described by one office staff member: *“When they rolled it out … there were issues with them being able to create the common record. And, it was just stopped”* (Participant #5).

Further hindering adoption was the patchwork of processes clinics used to manage PAs. As one pharmacist, referring to the common record stated: *“[It] really depends on the clinic...not everyone’s doing the same thing … it’s added a lot of work to my daily routine”* (Participant #24). This baseline lack of standardized processes coupled with a lack of training, for both the common record and auto-referral process, led to waste and duplicate work, which outweighed potential benefits and prevented widespread adoption. For example, participants described having to look in multiple places for information *(“We have to play Sherlock Holmes. We have to look at it. It’s not all in the same place”* (Participant #5)), the exact opposite of the intent of the common record and this led to wasted time: *“I’m having to read the chart, go through what I’m supposed to do with this, who did drop the ball on something, and by that time, it’s minutes of my time … it just wasted my time”* (Participant #18). Similar problems manifested for the auto-referral process with participants describing how poor communication about the auto-referral initiative led to confusion among clinic staff: *“clinics are coming back to us confus[ed] of where the medication is truly coming from”* (Participant #2).

### Feasibility

As indicated above, adoption was hindered by the poor feasibility of the initiatives due to the duplicative work and confusion that, in part, were caused by inadequate training and communication around the initiatives. As highlighted by a PA staff member: “*… we’re doing duplication of work. Where, you know, [specialty pharmacy] is getting the requests via a prescription and we’re also getting the request via telephone encounter from the clinic. So, like I said, it could end [up] being duplication of work where we’re getting both a medical and a prescription authorization”* (Participant #2). This lack of feasibility was also impacted by the lack of standardized workflow across clinics: *“… not everybody’s doing the same thing. So, with rheumatology, derm[atology], gastro[enterology], almost everything besides oncology, the referrals come to [specialty pharmacy], come to me when the medication has been approved. It’s added a lot of work to my daily routine”* (Participant #24).

### Future adaptations

Participants had many suggestions for improving the process for PAs within the healthcare system in general and in relation to the implemented initiatives. We focused only on those suggestions about the initiatives including improving standardization, transparency, and automation. Participants desired a standardized form for PA across insurance providers, but also wanted a workflow that minimized duplication and was consistent across Geisinger, which would help facilitate transparency and reduce workload. For example, one physician described how a standardized process and a transparency around where a request was in the PA process could then be communicated to patients and reduce follow-up calls. Participants also described wanting a system where it is clear at the point of care which benefit the medication should be billed under, whether it is covered by insurance, and what the PA requirements are. Finally, participants discussed wanting more automation to increase efficiency and reduce errors*: “… I think more automation … that human has to free text all this information, when really all that information could be on an order. I think that would really help not only staffing issues but human errors”* (Participant #2).

## Discussion

We found that participants overall felt the idea behind the initiatives was acceptable, however in practice, several challenges, such as lack of a standardized workflow for PA across clinics and ineffective training, impaired the feasibility and adoption of the common record and auto-referral initiatives. The baseline lack of a standardized PA process led clinics to adapt the initiatives to fit their own workflow which caused duplicative work downstream. Future initiatives should consider how process will be adopted and adapted by users and design the implementation accordingly.

While several studies have examined initiatives to improve the PA process [3, 11–14], this study is the first to examine the implementation of an auto-referral process. To our knowledge, our study is also the first to specifically examine implementation outcomes associated with initiatives to improve the PA process. While the findings from this study are directly applicable to the PA process in our healthcare system in the United States, our findings regarding the implementation outcomes may apply to other systemwide initiatives beyond PA that might be applicable for other countries.

Our study highlighted gaps including the need for initial and continued training, standardization of workflow, and the importance of not creating more work. That gaps can be addressed in future studies by deliberately using the science of implementation. For example, the pragmatic robust implementation and sustainability model accounts for both internal and external context factors which are important determinants of implementation and mirror the challenges we identified in our study [15].

### Strengths and limitations

While the themes we heard from participants were similar, our chief limitation is a small sample, specifically within the different roles (e.g., we only interviewed one office staff member) which may have affected the saturation of our data. Further interviews may have provided additional perspectives and a richer understanding of the implementation processes around the two initiatives we examined. Additionally, we did not interview any members of the implementation team responsible for developing and implementing the initiatives. Interviewing this group may have illuminated additional barriers or challenges associated with the initiatives. Patients were not selected for interviews because these system-level process initiatives were not patient facing.

## Conclusion

The acceptability, adoption, and feasibility of two initiatives to improve the PA process was limited due to implementation challenges such as ineffective training and an inverse relationship between adaptability and feasibility. Studies should examine the tradeoff between the adaptability and feasibility of initiatives. Future initiatives should proactively utilize the science of implementation to identify and address potential barriers to implementation.

## Supplementary Information

**Additional file 1.**

## Data Availability

All data generated or analyzed during this study are included in this published article and its supplementary information files.
