# Supplementary material for: Evaluating implementation outcomes (acceptability, adoption, and feasibility) of two initiatives to improve the medication prior authorization process
Source: BMC Health Serv Res. 2021 Nov 20;21:1259. doi: 10.1186/s12913-021-07287-2 (PMC8606066; doi:10.1186/s12913-021-07287-2)
Supplement: Supplementary file 1 — Additional file 1. [file 12913_2021_7287_MOESM1_ESM.docx]

Supplemental File:

Acceptability, appropriateness, and feasibility of two initiatives to improve the medication prior authorization process

AUTHORS: Laney K. Jones, PharmD, MPH, Ilene G. Ladd, MS, Christina Gregor, BS, Michael A. Evans, BSPharm, Jove Graham, PhD, Michael R. Gionfriddo, PharmD, PhD

**Supplemental file 1: Medical Staff Interview Guide**

**Medical Staff Interview Guide**

**Go through Information Sheet – Interview first**

*[self-introduction]- hello my name is [name], I am a [position]with Geisinger*

*Thank you for agreeing to take part in this study. As a reminder, your participation in this study is voluntary. We expect our discussion to last no longer than a half an hour. Our goal is to understand your experience with the medication prior authorization process.*

*Before we start, I would like to remind you that there are no right or wrong answers. We are interested in knowing what you think, so please feel free to be honest and to share your point of view. It is very important that we hear your opinions. We will record today’s conversation but we will not include your identifying information when we write up our report.*

*I would like to start the recording now; would that be ok? [start recorder]*

**Start Recorder**

*Our first set of questions are to provide some basic demographic information*

1. Position/title:

2. Dept (interviewer complete ahead of time)_________________

3. How long in position (years)

4. How long in organization (years)

5. Training/degree

6. Sex – interviewer should fill this in if possible _____________

**Interview questions:**

1. Describe your role in the prior authorization process.

2. What barriers do you currently experience with the prior authorization process?

*Geisinger recently implemented an intervention to improve the prior authorization process. This intervention aimed to improve transparency of prior authorization. There are 2 pieces to the intervention. The common record was developed in the electronic health record to allow for a single source of documentation for everything related to prior authorization approval such as prior authorization decision and cost data. Additionally, certain specialty medications are now being auto-routed to CareSite Specialty Pharmacy.*

*First, I’d like to ask you about the common record*

3. Were you aware of this intervention? (common record)

If no to common record, skip to question #14-15

If yes…

4. Let’s think about the common record; How was this introduced to your work unit?

a. When was the **common record** introduced?

b. How were you made aware of the **common record**?

c. What kind of training did you receive?

5. Since the implementation of the **common record**, what changes have you noticed to your workflow or process?

a. How do you use **common record** in your setting?

6. Do you think the **common record** has had a positive or negative impact?

a. In what way was this change positive or negative

7. What barriers has the **common record** alleviated?

8. What changes would you make to the **common record**?

Now, Let’s think about the **specialty medications being auto-routed to CareSite Specialty Pharmacy**;

9. Were you aware of this intervention (specialty medications)?

If no to specialty medications, skip to #16-17

If yes…How was this introduced to your work unit?

a. When was the **specialty medications being auto-routed to CareSite Specialty Pharmacy** introduced?

b. How were you made aware of the **specialty medications being auto-routed to CareSite Specialty Pharmacy**?

c. What kind of training did you receive?

10. Since the implementation of the specialty medications being auto-routed to CareSite Specialty Pharmacy, what changes have you noticed to your workflow or process?

a. How do you use **specialty medications being auto-routed to CareSite Specialty Pharmacy** in your setting?

11. Do you think the **specialty medications being auto-routed to CareSite Specialty Pharmacy** has had a positive or negative impact?

a. In what way was this change positive or negative?

12. What barriers has the **specialty medications being auto-routed to CareSite Specialty Pharmacy** alleviated?

13. What changes would you make to the **specialty medications being auto-routed to CareSite Specialty Pharmacy**?

Questions 14-15 are only for those who answered No to Q3.

14. What are your views about the **common record** as described?

15. Do you believe that the **common record** will help the prior authorization process? Why? Or Why not?

Questions 16-17 are only for those who answered No to Q9.

16. What are your views about the **specialty medications being auto-routed to CareSite Specialty Pharmacy** as described?

17. Do you believe that the **specialty medications being auto-routed to CareSite Specialty Pharmacy** will help the prior authorization process? Why? Or Why not?

Question 18 is for all interviewees

18. What suggestions do you have for the entire prior authorization process?

[closing]- that’s all we have for today unless you have anything else you would like to add about Prior Authorization. (pause for their response)

Thank you very much for sharing with us today!
